# *Aster koraiensis* Extract and Chlorogenic Acid Inhibit Retinal Angiogenesis in a Mouse Model of Oxygen-Induced Retinopathy

**DOI:** 10.1155/2018/6402650

**Published:** 2018-04-22

**Authors:** Junghyun Kim, Yun Mi Lee, Wookwon Jung, Su-Bin Park, Chan-Sik Kim, Jin Sook Kim

**Affiliations:** ^1^Korean Medicine Convergence Research Division, Korea Institute of Oriental Medicine, Daejeon, Republic of Korea; ^2^Department of Oral Pathology, School of Dentistry, Chonbuk National University, Jeonju 54896, Republic of Korea

## Abstract

*Aster koraiensis* extract (AKE) is a standard dietary herbal supplement. Chlorogenic acid (CA) is the major compound present in AKE. Retinal neovascularization is a common pathophysiology of retinopathy of prematurity, diabetic retinopathy, and wet form age-related macular degeneration. In this study, we aimed to evaluate the effects of AKE and CA on retinal neovascularization in a mouse model of oxygen-induced retinopathy (OIR). Vascular endothelial growth factor- (VEGF-) induced tube formation was assayed in human vascular endothelial cells. Experimental retinal neovascularization was induced by exposing C57BL/6 mice to 75% oxygen on postnatal day 7 (P7) and then returning them to normal oxygen pressure on P12. AKE (25 and 50 mg/kg/day) and CA (25 and 50 mg/kg/day) were administered intraperitoneally for 5 days (P12–P16). Retinal flat mounts were prepared to measure the extent of retinal neovascularization at P17. The incubation of human vascular endothelial cells with AKE and CA (1–10 *μ*g/mL) resulted in the inhibition of VEGF-mediated tube formation in a dose-dependent manner. The neovascular area was significantly smaller in AKE or CA-treated mice than in the vehicle-treated mice. These results suggest that AKE is a potent antiangiogenic agent and that its antiangiogenic activity may, in part, be attributable to the bioactive component CA.

## 1. Introduction

Retinal neovascularization is the most common cause of irreversible vision loss in individuals older than 65 years [1] and is a severe complication of retinopathy of prematurity, diabetic retinopathy, and wet form age-related macular degeneration (AMD) [2].

Vascular endothelial growth factor (VEGF) is a well-known proangiogenic and vascular permeability factor, and is a key mediator in the pathogenesis of these retinal diseases [3]. Recently, the use of VEGF antagonists to inhibit the VEGF signaling pathway has successfully diminished retinal neovascularization in several experimental animal models [4] and human subjects [5]. In numerous clinical trials, intravitreally injected anti-VEGF agents, including bevacizumab, ranibizumab, and aflibercept, notably suppressed neovascularization and stabilized vision loss [6–8]. However, the intravitreal injection of anti-VEGF agents presents the risk of adverse events [9, 10]. Repeated intravitreal injections increased the incidence of ocular complications, including endophthalmitis, ocular inflammation, traumatic cataracts, intraocular pressure elevation, retinal detachment, and vitreous hemorrhage [11]. Thus, interest in the use of oral agents has been increasing [12–14].

*Aster koraiensis* (Korean starwort) is a valuable perennial plant native to Korea. This herb has been used as food, such as appetizers and side dishes, and in traditional medicine to treat several diseases, including pneumonia, chronic bronchitis, diabetes, and pertussis [15, 16]. In our prior studies, we reported that the extract of* A. koraiensis* (AKE) prevented podocyte apoptosis in the renal tissues of streptozotocin- (STZ-) induced diabetic rats [17] and also inhibited retinal pericyte apoptosis in these rats [18]. Although various effects of AKE on retinal injury in an animal model of diabetes have been reported, its effect on retinal pathogenic neovascularization remains unknown. To elucidate this, we investigated the inhibitory effect of AKE and its major compound, chlorogenic acid (CA), on retinal neovascularization in a mouse model of oxygen-induced retinopathy (OIR). We also investigated the inhibitory effect of AKE and CA on the VEGF-induced tube formation of human vascular endothelial cells.

## 2. Materials and Methods

### 2.1. Preparation of AKE

The aerial parts, which include the flowers, leaves, and stems, of* A. koraiensis* were purchased from Gongju (Chungcheongnam, South Korea) in August 2007. The AKE was prepared according to a previously reported method [17]. Briefly, 2.5 kg* A. koraiensis *was accurately weighed and extracted with EtOH (3 × 20 L) via maceration at room temperature for 3 days. The extracted solution was concentrated in vacuo at 40°C to provide an AKE powder (303 g). Voucher specimens of* A. koraiensis *were deposited at the Herbarium of the Korea Institute of Oriental Medicine (Daejeon, Korea; Herbarium number KIOM-83A). AKE was standardized, using CA as a reference compound (Sigma-Aldrich; Merck Millipore, Darmstadt, Germany), by high-performance liquid chromatography (HPLC). The HPLC fingerprint and the CA content of AKE are described in our previous report [18].

### 2.2. Cell Viability Assay

Cell viability was examined using an MTS assay kit (CellTiter 96® AQueous One Solution Cell Proliferation Assay; Promega Corporation, Madison, WI, USA). Human umbilical vein endothelial cells (Korean Cell Line Bank, Seoul, Korea) were plated (1 × 10^4^ cells/well) in quadruplicate into 96-well plates containing various doses of AKE or CA (1–100 *μ*g/mL). Cell viability was measured at 24 h following incubation. The results of the MTS assay were obtained by measuring absorbance using a microplate reader (Tecan Group Ltd., Männedorf, Switzerland) at 490 nm. All experiments were repeated three times.

### 2.3. Tube Formation Assay

Tissue culture plates (96 wells) were coated with 400 *μ*L growth factor reduced basement membrane matrix (Matrigel®; BD Biosciences, Franklin Lakes, NJ, USA). Human umbilical vein endothelial cells were seeded at a density of 1 × 10^6^ cells/well and treated with serum-free EGM™-2 media (WelGENE, Inc., Daegu, Korea) containing AKE or CA (0–10 *μ*g/mL) and recombinant human VEGF (20 ng/mL) for 17 h at 37°C. Capillary-like tube structures formed by human umbilical vein endothelial cells on the Matrigel were photographed with a DP71 digital camera (Olympus Corporation). Tube formation was quantified by counting the number of branching points of the capillary-like structures per visual field. The experiments were repeated three times independently.

### 2.4. Experimental Retinal Neovascularization in OIR

Experimental retinal neovascularization was induced in C57BL/6 mice, as previously described [19]. Mice were exposed to 75% oxygen on postnatal day 7 (P7) and then returned to normal oxygen pressure on P12. The mice were divided into five groups of seven mice each as follows: (1) OIR mice; (2) OIR mice treated with AKE (25 mg/kg body weight); (3) OIR mice treated with AKE (50 mg/kg body weight); (4) OIR mice treated with CA (25 mg/kg body weight); and (5) OIR mice treated with CA (50 mg/kg body weight). AKE and CA were administered intraperitoneally for 5 days (P12–P16). The mice in the OIR group received an equal volume of the vehicle for 5 days. All procedures were approved by the Institutional Animal Care and Use Committee (IACUC; approval number 14-053).

### 2.5. Fluorescein-Dextran Microscopy and Lectin Staining for Neovascular Area Analysis

At necropsy (P17), all mice were anesthetized by isoflurane inhalation. Fluorescein-dextran (FD40; Sigma-Aldrich; Merck Millipore) in PBS at a concentration of 50 mg/ml was injected into the left ventricle. The tracer dye was allowed to perfuse for 15 min and the eyeballs were then placed in 4% paraformaldehyde for 1.5 hours. The retinas were dissected and then mounted on microscope slides. The whole-mount retinas were observed using a fluorescence microscope (Olympus Corporation, Tokyo, Japan). The nonperfusion area in the retina was determined by ImageJ software (National Institutes of Health, Bethesda, MD, USA). The neovascular tufts in the retina were stained with rhodamine-labeled* Bandeiraea simplicifolia *isolectin B4 (Vector Laboratories Ltd., Burlingame, CA, USA). The neovascular areas labeled with lectin were examined using a fluorescence microscope. The sizes of the neovascular tufts were calculated using the ImageJ software.

### 2.6. Real-Time PCR Analysis

Frozen retinal samples were weighed and the total RNA was isolated using TRIzol® reagent (Invitrogen; Thermo Fisher Scientific, Inc., Waltham, MA, USA). Real-time PCR was conducted according to a previously described protocol [20]. The primer sequences were as follows: VEGF: 5′-TCC TCC TAT CTC CAC CAC CTA TCC-3′ and 5′-GAC CCA GCC AGC CAT ACC C-3′ and GAPDH: 5′-AAC GAC CCC TTC ATT GAC-3′ and 5′-TCC ACG ACA TAC TCA GCA C-3′. The mRNA levels of VEGF were determined using the iQ5 optical system software (Bio-Rad Laboratories, Inc., Hercules, CA, USA).

### 2.7. Statistical Analysis

Group data were analyzed by one-way analysis of variance followed by Tukey's multiple comparison test or an unpaired Student's* t*-test, using GraphPad Prism v.6.0 software (GraphPad Software, Inc., La Jolla, CA, USA). A *p* value of <0.05 was considered to indicate a statistically significant difference.

## 3. Results

### 3.1. HPLC Analysis of AKE

The content of the major constituent compound in AKE was determined via HPLC analysis. CA (1.24 ± 0.02%) was found to be the major component of AKE (Table 1).

### 3.2. AKE and CA Inhibit VEGF-Induced Tube Formation in Human Vascular Endothelial Cells

**Figure 1:**
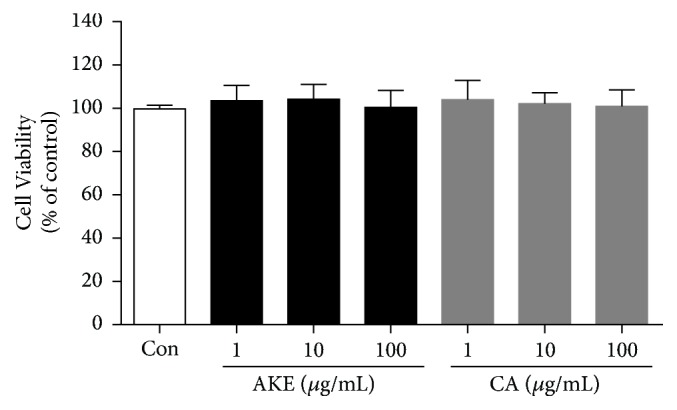
*Effects of AKE and CA on the viability of human vascular endothelial cells*. The viability of human vascular endothelial cells was determined by MTS assay. Data are expressed as percentage of control. Data are expressed as mean ± SEM; *n* = 4.

**Figure 2:**
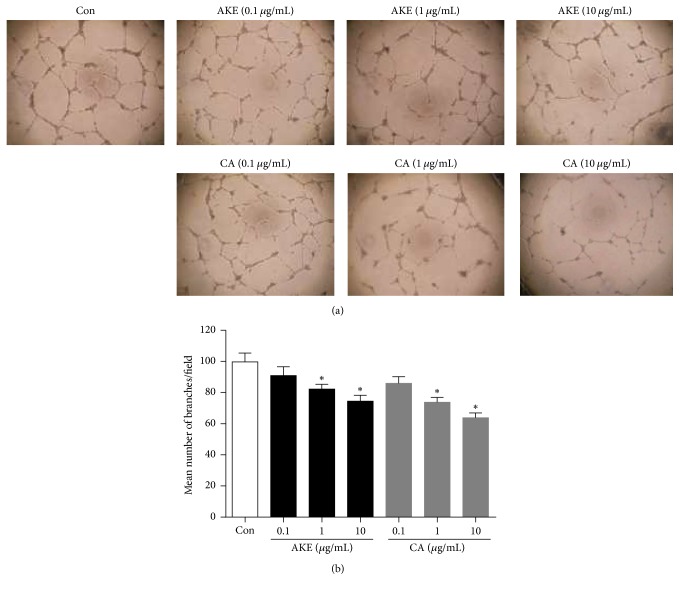
*AKE inhibits tube formation in human vascular endothelial cells*. (a) Human vascular endothelial cells were treated with serum-free media containing AKE or CA (0–10 *μ*g/mL) with recombinant human VEGF (20 ng/mL) for 17 h. Tube formation on Matrigel was observed with a microscope. (b) The bar graph represents the quantification of tube formation. Data are expressed as mean ± SEM, *n*  =  4, ^*∗*^*p* < 0.01 versus control.

To investigate the cytotoxic effect of AKE on human vascular endothelial cells, we performed an MTS assay using various concentrations of AKE or CA (1–100 *μ*g/mL). The viability of AKE or CA-treated human vascular endothelial cells was not affected up to concentrations of 100 *μ*g/mL (Figure 1). Next, we examined whether AKE or CA could inhibit tube formation, an endothelial function crucial to angiogenesis, in human vascular endothelial cells. VEGF was used as an angiogenic factor. Treatment with AKE or CA inhibited the formation of the extensive capillary-like networks of human vascular endothelial cells in a dose-dependent manner (Figure 2). The inhibitory activity of CA was more potent compared with that of AKE.

#### 3.2.1. AKE and CA Inhibit Retinal Neovascularization in OIR

**Figure 3:**
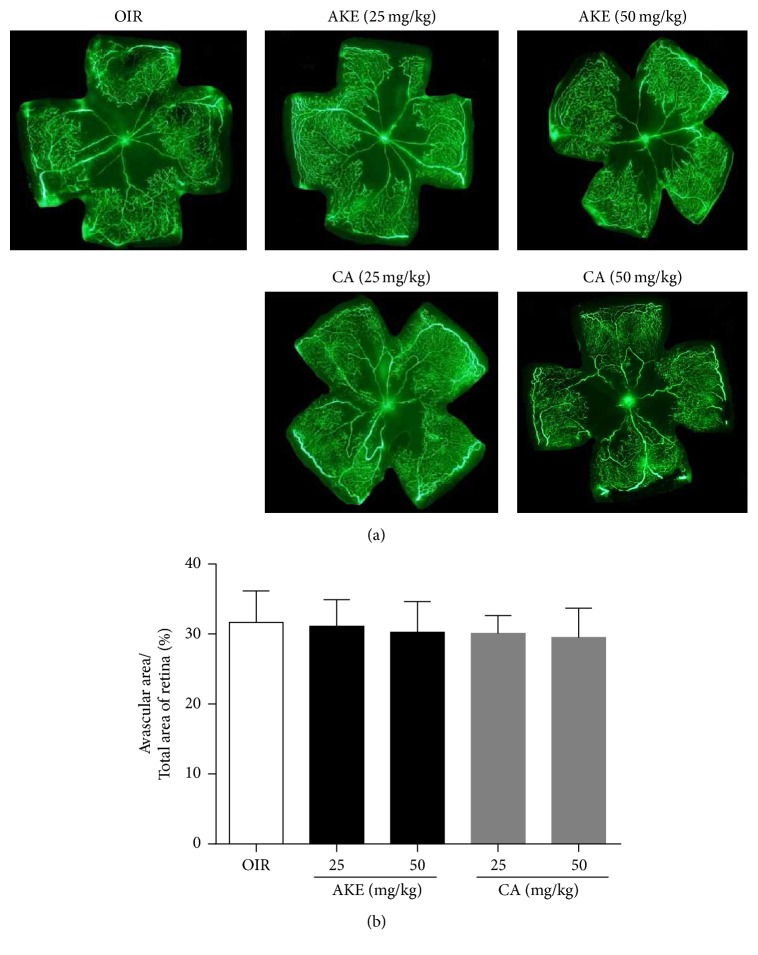
*The effect of AKE and CA on vascular obliteration of the central retina in OIR mice*. (a) The retinal blood vessels were visualized via fluorescein angiography using FITC-dextran. (b) The quantification results are expressed as the percentage of the central nonperfused area within the total retinal area. The bar graph values represent the mean ± SEM, *n* = 7, ^*∗*^*p* < 0.05 versus OIR mice.

**Figure 4:**
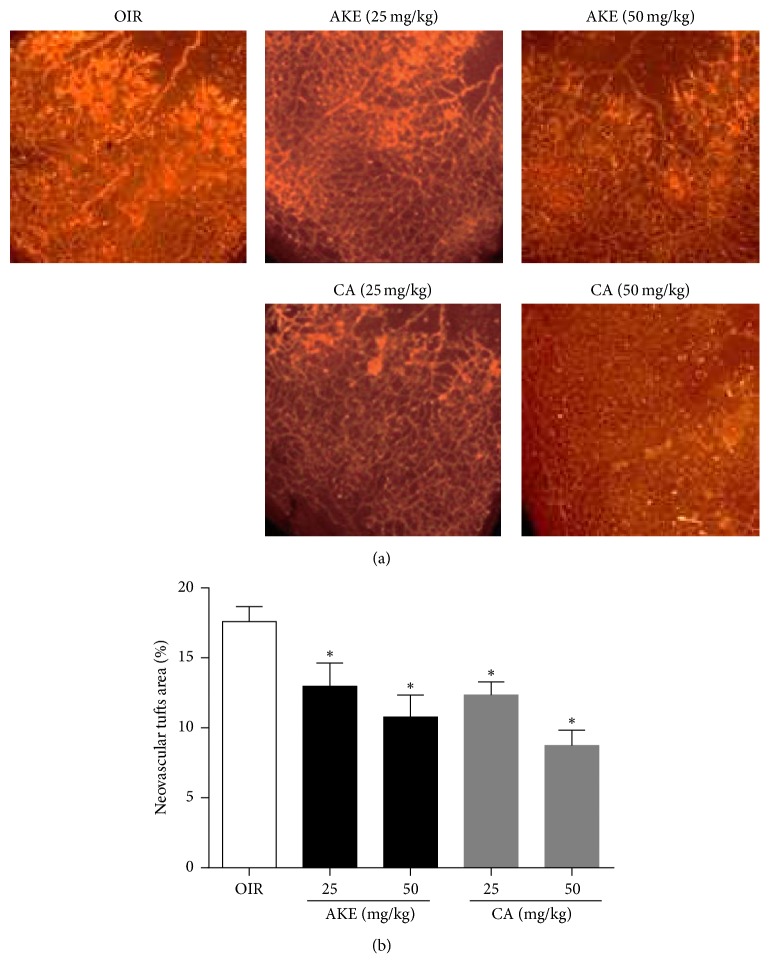
*The effect of AKE and CA on retinal neovascularization in OIR mice*. (a) The retinal neovascular tufts were visualized using isolectin B4 staining. (b) Quantification results are expressed as neovascular tufts on the retina surface. The bar graph values represent the mean ± SEM, *n* = 7, ^*∗*^*p* < 0.05 versus OIR mice.

The mice subjected to ischemic retinopathy showed vascular obliteration of the central retina and pathogenic retinal neovascularization. Newly formed neovascular tufts were visualized by immunofluorescence staining with isolectin B4. OIR mice treated with AKE or CA exhibited a significant decrease in these retinal vascular changes that occur during ischemic retinopathy. As presented in Figure 3, treatment with AKE or CA failed to induce significant changes in the vascular obliteration of the central retina. However, AKE inhibited the formation of neovascular tufts by 26.27 ± 4.24% and 38.75 ± 4.04% at doses of 25 and 50 mg/kg/day, respectively. CA also inhibited retinal neovascularization by 29.68 ± 2.35% and 50.24 ± 2.77% at doses of 25 and 50 mg/kg/day, respectively (Figure 4). These results indicated that AKE and CA treatments significantly reduce the size of neovascular tufts, demonstrating that CA is an antiangiogenic bioactive compound of AKE.

#### 3.2.2. AKE and CA Downregulate VEGF mRNA Expression

**Figure 5:**
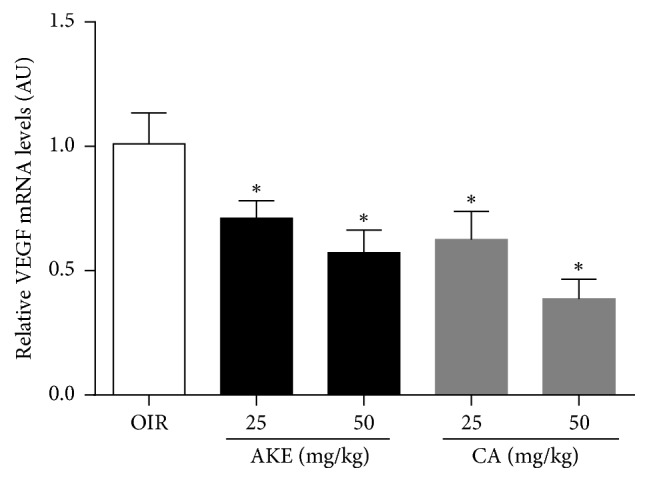
* The effect of AKE and CA on VEGF mRNA expression in OIR mice*. Real-time PCR analysis of VEGF mRNA levels in OIR mice. VEGF mRNAs were markedly reduced after AKE or CA treatment. The data are shown as the mean ± SEM, *n* = 7, ^*∗*^*p* < 0.05 versus OIR mice.

To examine the changes in VEGF expression in the retina, we measured the expression levels of VEGF mRNA using real-time PCR. As predicted, we observed a marked increase in VEGF mRNA during ischemic retinopathy. However, the VEGF mRNA levels markedly decreased following treatment with AKE or CA in the OIR mice (Figure 5).

## 4. Discussion

Pathogenic angiogenesis is a primary cause of severe vision loss in several retinal degenerative diseases, including diabetic retinopathy and wet form AMD [21]. VEGF and its receptors play an important role in the development of these retinal disorders [3], and inhibiting angiogenesis by targeting VEGF has become a major focus in drug development [22]. In the present study, we aimed to evaluate the effect of AKE on retinal neovascularization in a mouse model of OIR. To the best of our knowledge, this study demonstrated for the first time that AKE inhibits tube formation in human vascular endothelial cells* in vitro* through a VEGF-mediated mechanism. In addition, AKE significantly suppressed retinal neovascularization and VEGF mRNA expression in a mouse model of experimental OIR. Moreover, CA is one of the major compounds present in AKE; CA also exhibited a preventive effect against pathological retinal neovascularization. Taken together, these results suggest that the inhibitory effect of AKE on retinal neovascularization primarily stems from its potent anti-VEGF activity and that its antiangiogenic activity may, in part, be due to the bioactive compound CA.

VEGF is a potent angiogenic and vascular permeability factor [23] that stimulates endothelial cell proliferation and angiogenesis. In OIR mice, VEGF expression was suppressed during the hyperoxic phase (P7–P12) [24]. Once hyperoxia was terminated (P12–P17), hypoxia-driven upregulation of VEGF was observed even under normoxic conditions [25, 26]. Furthermore, in ischemic retinopathy, such as that present in diabetic retinopathy and neovascular AMD, the robust upregulation of proangiogenic VEGF expression leads to the activation of angiogenic signaling pathways and triggers neovascularization [27]. Numerous studies have suggested that VEGF has a key role in retinal vasculopathy, and its inhibition significantly blocks the pathogenic alterations of retinal vasculature [28, 29]. Anti-VEGF agents were recently reported to exhibit beneficial effects in patients with proliferative diabetic retinopathy and neovascular AMD [2, 30].

Medicinal herbs are rich sources of potential preventive and therapeutic agents. AKE is a standardized dietary herbal supplement. To the best of our knowledge, our study demonstrated the antiangiogenic effects of AKE* in vitro* and* in vivo* for the first time. Several studies have reported that certain crude herbal extracts and phytochemicals can inhibit pathogenic neovascularization in tumorigenesis [31, 32] and retinal neovascular diseases [33–37]. CA is the ester of caffeic acid with quinic acid, and a major phenolic compound in coffee and various fruits [38]. CA is also a major compound present in AKE [18]. This phytocompound has potent antioxidant [39], anti-inflammatory [40], and anticancer effects [41]. Kim et al. [42] reported that CA inhibited laser-induced choroidal neovascularization in a rat model of neovascular AMD. More recently, Park et al. [43] showed that CA blocks hypoxia-stimulated angiogenesis in human vascular endothelial cells through inhibition of the hypoxia-inducible factor-1*α*/AKT signaling pathway. In the present study, CA also inhibited the VEGF-mediated tube formation of human vascular endothelial cells and retinal neovascularization in mice with ischemic retinopathy. Although the underlying mechanism of action of AKE as a VEGF inhibitor remains unclear, it is hypothesized that the antiangiogenic activity of AKE may be due to the antiantigenic effect of the bioactive component CA

In conclusion, this is the first study to provide evidence that AKE and its bioactive compound, CA, inhibit experimental retinal neovascularization in ischemic retinopathy* in vivo*. In addition,* in vitro* studies showed that AKE and CA inhibit VEGF-induced tube formation in human vascular endothelial cells. Further studies may be required to determine the feasibility of using AKE for the treatment of patients with ischemic retinopathy.

## Figures and Tables

**Table 1:** Chlorogenic acid content in AKE.

Compound	Content (mean ± SD, *n* = 3)
	mg/g
Chlorogenic acid	12.35 ± 0.22
